# Building capacity for librarian support and addressing collaboration challenges by formalizing library systematic review services

**DOI:** 10.5195/jmla.2019.443

**Published:** 2019-07-01

**Authors:** Sandra McKeown, Amanda Ross-White

**Affiliations:** Health Sciences Librarian, Bracken Health Sciences Library, Queen’s University, Kingston, ON, K7L 3N6, Canada, sandra.mckeown@queensu.ca; Health Sciences Librarian, Bracken Health Sciences Library, Queen’s University, Kingston, ON, K7L 3N6, Canada, amanda.ross-white@queensu.ca

## Abstract

**Background:**

Many health sciences librarians are noticing an increase in demand for systematic review support. Developing a strategic approach to supporting systematic review activities can address commonly reported barriers and challenges including time factors, methodological issues, and supporting student-led projects.

**Case Presentation:**

This case report describes how a health sciences library at a mid-sized university developed and implemented a structured and defined systematic review service in order to build capacity for increased librarian support and to maximize librarians’ time and expertise. The process also revealed underlying collaboration challenges related to student-led systematic reviews and research quality concerns that needed to be addressed. The steps for developing a formal service included defining the librarian’s role and a library service model, building librarian expertise, developing documentation to guide librarians and patrons, piloting and revising the service model, marketing and promoting the service, and evaluating service usage.

**Conclusions:**

The two-tiered service model developed for advisory consultation and collaboration provides a framework for supporting systematic review activities that other libraries can adapt to meet their own needs. Librarian autonomy in deciding whether to collaborate on reviews based on defined and explicit considerations was crucial for maximizing librarians’ time and expertise and for promoting higher quality research. Monitoring service usage will be imperative for managing existing and future librarian workload. These data and tracking of research outputs from librarian collaborations may also be used to advocate for new librarian positions.

## BACKGROUND

As publication of systematic reviews continue to rise every year [1, 2], librarians continue to define their roles [3] and develop service models [4–7] for supporting these research activities. Barriers to supporting systematic reviews often relate to time factors [7–11], which can have implications for the librarian’s ability to obtain training and experience, to develop and deliver educational content, and to participate on review teams. Librarians have also reported a number of collaboration challenges related to methodological issues that affect the quality of reviews [10]. Most recently, librarians have been grappling with how best to support students conducting systematic reviews [10, 12, 13].

To address some of the universal barriers and challenges to providing this type of research suppport, librarians at Bracken Health Sciences Library, Queen’s University, in Kingston, Ontario, Canada, set out to develop a structured service for systematic review support. Like other health sciences librarians [4, 5, 7, 9, 13], we have noticed a substantial increase in the number of requests for systematic review support. New and growing graduate programs have been contributing factors, because research courses can include a systematic review assignment. We have also noticed an influx of medical residents and even medical students and other undergraduate students contacting us for support. Requests from faculty to have a librarian conduct systematic review searches have been increasing as well.

Expanding and improving our research support services aligns with the university’s and library’s strategic priority to strengthen our research prominence [14, 15]. University-wide objectives for meeting this goal include increasing research support, improving intra- and inter-faculty and cross-university collaboration, and increasing and improving our impact through high-quality publications [15].

## STUDY PURPOSE

In response to increasing requests for systematic review support, we developed and implemented a structured and defined service for supporting systematic reviews. The purpose of developing a formalized service was to build capacity for increased librarian support and to maximize librarians’ time and expertise in providing this support.

## CASE PRESENTATION

### Setting

Queen’s University is a research-intensive institution with five faculties: Arts and Science, Education, Engineering and Applied Science, Law, and Health Sciences. The library system supports more than 24,000 students and 3,500 faculty and researchers and includes 5 faculty library locations [16]. Six librarians at Bracken Health Sciences Library (including a department head) support just over 2,000 faculty and 3,000 students from the Faculty of Health Sciences [17]. The faculty includes the Schools of Medicine, Nursing, and Rehabilitation Therapy as well as departments of life sciences and biochemistry, a bachelor of health sciences program, and specialized graduate programs in aging and health, health care quality, and public health sciences. In January 2016, librarians at Bracken began taking steps to strategically plan and implement a structured systematic review service.

### Defining the librarian’s role and a library service model

We conducted a literature search to review the various systematic review activities that involve librarians [6, 18–21] and to guide development and management of library systematic review services [6, 8–10, 22]. We also searched the Internet for systematic review services offered by peer libraries to generate additional ideas for the service and to use for benchmarking purposes. Reviewing information on library websites was a particularly helpful exercise and prompted us to create a tiered service model in order to differentiate between providing guidance or instruction and participating as a collaborator on a systematic review team.

Three hour-long planning meetings took place between April and May 2016 to conceptualize and develop a service model. In addition to the health sciences librarians, one of the librarians from the library for humanities and social sciences was invited and attended some of these meetings. The health sciences librarians work closely with the liaison librarian for the School of Kinesiology and Health Studies (part of the Faculty of Arts and Science) to coordinate and deliver systematic review support. While technically outside of the Faculty of Health Sciences, graduate students in kinesiology and health studies sometimes approach or are referred to librarians at Bracken for support. For example, it often makes sense for these students to include medical databases such as Ovid MEDLINE as part of their searches. In such cases, their liaison librarians provides support for the sociology and humanities databases and then refers students to the health sciences librarians for support with health databases.

During the planning meetings, we considered the systematic review support that we had provided in the past and any other types of support we or our patrons might like to see offered in future. Acknowledging that we would not have the resources to “do it all” meant that certain levels of support could not be guaranteed as part of a core service. For example, the nursing librarian had participated in screening studies for eligibility, but we agreed that this would not be part of the core service because it can be incredibly time-consuming and librarians might not have the subject expertise. It was important to us to continue having the autonomy to exercise discretion in offering additional levels of support above and beyond any core services.

We decided on a two-tiered service model for Bracken that would offer systematic review support in the form of advisory consultation or collaboration (Table 1). Advisory consultation would be the core service available to all faculty, staff, and students and would be largely educational. The main activities would involve advising patrons about the review process, research question formulation, resource selection, search methods, and citation management.

**Table 1:** Librarian activities involved in advisory consultation and collaboration

	Advisory consultation	Collaboration
Advise on a preliminary search to determine if a review or protocol on the same topic already exists	✓	✓
Help with formulating or refining the review question	✓	✓
Advise on review steps	✓	✓
Recommend databases and resources to search	✓	✓
Advise or instruct on database or resource-specific search methods and techniques	✓	✓
Advise or instruct on setting up search alerts for new publications	✓	✓
Advise or instruct on citation management or review software	✓	✓
Advise on search methods for locating grey literature	✓	✓
Advise on additional methods for locating studies (e.g., searching trial registries, hand-searching, searching cited references)	✓	✓
Advise on how the search methods should be reported for transparency and reproducibility	✓	✓
Conduct a preliminary search to determine if a review or protocol on the same topic already exists		✓
Develop and execute database- or resource-specific search strategies		✓
Set up search alerts for new publications		✓
Document database-specific search strategies for transparency and reproducibility		✓
Export search results into desired format (e.g., Excel spreadsheet, text or RIS file)		✓
Import search results to citation management or review software		✓
Assist with search methods for locating grey literature		✓
Assist with additional methods for locating studies (e.g., cited reference searching)		✓
Remove duplicate search results		✓
Write up the search methods according to PRISMA or other appropriate guidelines		✓

In addition to advisory consultation, librarian participation on review teams as collaborators would be available for faculty or review teams including faculty. The main activities would involve designing, executing, and documenting database searches; providing references in the preferred format or exporting them into the preferred software; and writing up the search methods. Since librarian collaboration can meet the criteria for authorship [23], librarians are encouraged to discuss coauthorship when they negotiate collaboration. Librarians maintain the autonomy to decline requests to collaborate. In such cases, advisory consultation would be offered.

The health sciences librarians decided to share requests for systematic review support outside of their own liaison roles to enable a more even distribution of workload. Regardless of how the requests are made and received, if librarians are unable to respond to or meet with the requestors in a reasonable amount of time, they can ask the other health sciences librarians for assistance.

### Building librarian expertise

Prior to developing a formal service at Bracken, three of the six health sciences librarians provided systematic review support. This included delivering curriculum-integrated instruction about conducting systematic reviews to graduate students in research courses, meeting with students and medical residents about their specific review topics, and participating as collaborators on faculty review teams. To help balance librarian workload distribution amidst the increasing demand for systematic review support, we proposed involving the other health sciences librarians in providing support. The other librarians agreed, with support from the department head.

Supporting systematic reviews can be intimidating for inexperienced and experienced librarians alike. The three health sciences librarians with experience in providing systematic review support mentored the other librarians by inviting them to observe consultations and offering to review and discuss their systematic review work. All librarians regularly discuss their search strategies with one another or formally peer review each other’s searches using the PRESS checklist [24].

Capitalizing on the ability to learn from one another, we organized two one-and-a-half-hour group training sessions in 2017 to share knowledge and discuss strategies for providing systematic review support, such as best practices for limiting database searches to human studies or adults only. Beginning in 2018, we try to meet for at least one hour each month to discuss systematic review successes and challenges, lessons learned, and journal articles of interest.

Other strategies to help develop and maintain the knowledge and skills necessary to support systematic reviews have been encouraged, such as joining relevant groups and email discussion lists (e.g., the Medical Library Association’s [MLA’s] Systematic Reviews Special Interest Group). Additionally, group viewings of continuing eduation webinars are organized and promoted to all Queen’s librarians, given that systematic reviews are also conducted in disciplines outside of the health sciences, such as in engineering, geography, psychology, and education [20–23]. Librarians supporting these faculties provide research review support as part of their reference service, similar to the advisory consultations provided by the health sciences librarians. Some of these librarians are also seeking to expand their knowledge and level of involvement in supporting systematic reviews. Librarians can also use their individual professional development funds to develop their knowledge in this area.

### Creating documentation to guide librarians and patrons

We created a work plan document incorporating feedback from the other health sciences librarians to facilitate meetings with researchers (supplemental appendix). The work plan was designed to guide librarians through the initial meeting, prompting us to ask pertinent questions about the researchers and reviews that can be recorded and called upon later. Similar to work plans found on the websites of other libraries that offer systematic review support, the document asks questions about the research question, study parameters, search methods for identifying eligible studies, citation management, and other aspects of the review. The final page describes the two-tiered service model, listing potential librarian roles for advisory consultation and collaboration to better prepare librarians for discussions about their role and possible coauthorship.

A LibGuide for systematic reviews was developed to compile information that advises researchers and librarians who are engaged in systematic review activities. In addition to faciliating access to essential handbooks, guidelines, articles, websites, and tools, the guide provides information about review protocols, comprehensive searching, critical appraisal of studies, reporting standards, citation management, and review software. Information about library support and the work plan document is also available from the LibGuide. When researchers inquire about systematic review support, librarians can email them a link to the LibGuide and encourage them to peruse the content.

### Piloting and revising the service model

The two-tiered service model was piloted for a full year from May 2016 until April 2017. The health sciences librarians met four times during this period to discuss challenges and opportunities for improvement. Since then, we have continued to check in periodically to make further revisions to the service as necessary.

Some revisions to the service have been relatively straightforward to address, such as deciding to broaden the service to include other review types. In meeting with patrons who requested “systematic review” support, it was evident that the systematic review sevice would benefit from librarian knowledge of other review types. Consistent with other librarians’ accounts [6, 7], prospective researchers at Queen’s often presented with research questions or methods that were less suitable for a traditional systematic review. This provided librarians the opportunity to educate patrons about various review methodologies that might be more appropriate for their purposes, many of which also required systematic searching. As such, the scope of the service was broadened to encompass other review types that went beyond a standard literature review. Indeed, the journal *Systematic Reviews* took the position that new forms of reviews such as scoping reviews, rapid reviews, and evidence maps are “all in the family” of systematic reviews [25].

Other revisions to the service are complex and require continual monitoring to manage increases in service uptake, such as deciding when to collaborate on review teams. Supporting review teams that include faculty but are led by medical residents or other students can be problematic for a number of reasons [9, 10]. It is not uncommon for residents or other graduate students to lead systematic reviews and other review types for the purpose of publication without any prior experience in conducting this type of research. Faculty do not generally attend meetings with the librarian and often seem somewhat removed from the research process in such cases. As a result, these reviews do not always have a well-defined question or objectives, and the researchers often underestimate the amount of time and effort involved. This may explain why some of these reviews are discontinued or never submitted for publication after the librarian has invested a significant amount of their time.

To better utilize our time and maximize our impact, we determined that it was crucial to gauge the probable quality of research reviews and the likelihood of the research being published before agreeing to collaborate on review teams that include faculty, whether student-led or faculty-led. We now consider a number of characteristics about the review in question before deciding whether to collaborate (Table 2), including but not limited to quality indictors such as adherence to best practices. The decision to collaborate is informed by considering these criteria collectively and, in some cases, the review team may be willing and able to address some of these concerns. Because every review is unique, the characteristics that librarians consider vary in significance for each review. For example, if researchers are conducting a Cochrane review, having a research question that might not identify any eligible studies for inclusion [26] can be less problematic for publication than if researchers are conducting a non-Cochrane review and plan to submit to a journal.

**Table 2:** Review characteristics for librarian collaboration consideration

Review characteristics
Is the review being conducted under the auspices of a systematic review collaboration (e.g., Cochrane, JBI, Campbell)?
Has a simliar review already been published recently?
Can the researcher(s) clearly describe the research question?
Has the researcher(s) established inclusion and exclusion criteria?
Does the research question seem manageable in scope (not likely to yield too many eligible studies)?
Does the research question seem worthwhile (not likely to yield no or too few eligible studies)?
Does the review type match the research purpose?
Can the researcher(s) clearly describe the rationale and planned methods of the review?
Has a protocol been prepared?
Does the review team plan to follow best practice standards, such as PRISMA?
Does the review team agree to a comprehensive search approach (e.g., searching all key databases, employing relatively broad search strategies)?
Will the screening process involve the decision of two screeners for each item reviewed (at the citation or abstract level and full-text level)?
Does the research project seem manageable for the number of review team members?
Are the review timelines realistic and feasible?

### Marketing and promoting the service

We advertised the service in several ways, including an annoucement in the Faculty of Health Sciences newsletter, posts made on social media, and ongoing mentions at student library orientations and resident research day. Presentations have been made to faculty at Academic Council and other meetings and to the medical residency program directors and research coordinators. Stakeholders, including University Research Services and our librarian colleagues in other disciplines, have been informed about the service and encouraged to make referrals. The new LibGuide, which is visible on other health sciences LibGuides and library pages, also promotes the service. Concerns about promoting the service leading to overwhelming demand were mitigated by a slow roll out of promotional activity. This allowed us time to revise the parameters of the service as needed.

### Evaluating service usage

The LibGuide for systematic reviews went live in July 2016 and received 435 views by the end of the year. The guide continues to gain traction with 2,400 views in 2017 and more than 4,500 views in 2018. Ongoing content revisions and updates keep the guide current and relevant.

Advisory consultations are tracked with the same library widget used to track other educational consultations, which includes a notes field. The notes section specifically mentions providing support for systematic reviews or other research reviews on seventeen occasions in 2015, forty in 2016, thirty-five in 2017, and seventy-four in 2018, ranging from approximately thirty minutes to two and a half hours in length. In some cases, researchers met with a librarian more than once. However, these numbers may significantly underestimate the actual number of advisory consultations. Librarians revealed on more than one occasion that they did not remember being asked to indicate which educational consultations were for research review support in order to track service uptake.

**Figure 1:**
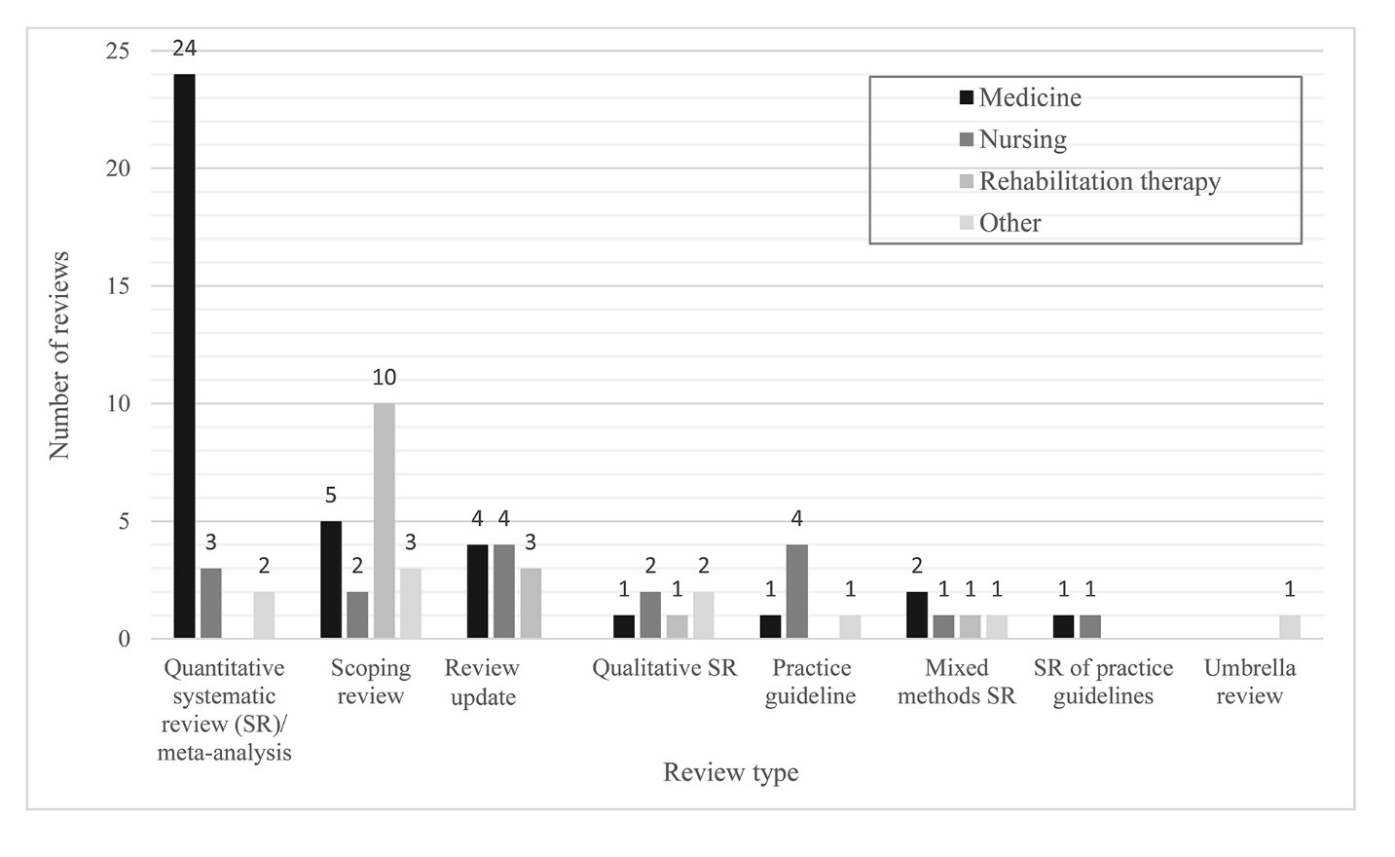
Librarian research review collaborations in 2017/18 by requester affiliation

An Excel spreadsheet was created to track librarian support in the form of collaboration for systematic reviews and other research reviews from 2017 onward. The number of collaborations increased from 32 in 2017 to 48 in 2018. The majority of total collaborations were with medicine (48%, 38/80), followed by nursing (21%, 17/80) and rehabilitation therapy (19%, 15/80). The remaining collaborations (13%, 10/80) were with other departments, programs, and schools such as life sciences and biochemistry, public health sciences, health sciences education, and kinesiology and health studies. The majority of librarian collaborations were for quantitative systematic reviews with or without meta-analysis (36%, 29/80), followed by scoping reviews (25%, 20/80) and updates for previous research reviews of any kind (14%, 11/80) (Figure 1). Other librarian collaborations were for qualitative systematic reviews (8%, 6/80), practice guidelines (8%, 6/80), mixed methods systematic reviews (6%, 5/80), systematic reviews of practice guidelines (3%, 2/80), and an umbrella review (1%, 1/80).

While all six health sciences librarians have been involved in providing advisory consultation, the department head may refer requests for collaboration because they have less time available. The service usage reported here does not take into account reference questions that we receive regularly from researchers before, after, or in place of advisory consultation or collaboration, including specific questions about citation management and inquiries about systematic review software. It also does not capture curriculum-integrated instruction surrounding research reviews, which has increased and expanded from graduate to undergraduate programs.

## DISCUSSION

Formalizing our systematic review service better positioned us to meet the increased demand for librarian support by distributing the work among the team of health sciences librarians. It also allowed us to address collaboration challenges related to student-led systematic reviews and research quality concerns by implementing a more judicious approach. Other librarians have reported limiting collaboration when the research plan is not well organized [10]. Our case report offers librarians an explicit list of review characteristics to consider when deciding whether or not to participate on a review team. We recognize that offering collaboration on a case-by-case basis might not provide a consistent experience for all patrons. However, exercising prudence in collaboration decisions is critical if librarians are to advocate against what has been called “the mass production of unnecessary, misleading, and conflicted systematic reviews and meta-analyses” [2].

While we do not require researchers to complete a protocol for collaboration as other librarians have reported [4, 9, 10], completing the work plan document with researchers helps us gauge how prepared and organized they are. In this way, the work plan can help us decide whether to collaborate on a review. Even though decisions not to collaborate can still result in advisory consultation, we suspect that this approach reduces the overall amount of time librarians spend providing support.

It will be imperative to continue monitoring service uptake and seek new efficiencies to help predict and manage future demand. Although we have been able to meet the rising demand for systematic review support to date, this might not always be the case. We do not currently charge for advisory consultation or collaboration, but some libraries have found it necessary to start a fee-based service for participating on review teams [7]. Other strategies reported to help meet growing demand for support include lobbying for more librarian positions [9]. Tracking and reporting service uptake may help us advocate for a new health sciences librarian position eventually. Finding an efficient and accurate method for tracking service uptake has proved difficult as we try to build an approach with our preexisting methods for tracking statistics, which are not generally detailed or designed to track work that can take place over long periods of time. Tracking which, and how many, librarian collaborations lead to formal publications can also help inform and advocate for the future direction of this library service.

Librarian involvement in systematic reviews shows no sign of slowing down any time soon. Growing research evidence shows that librarian involvement in systematic reviews is a proven way to improve the quality of research reviews [27–30]. Prominent medical journals encourage researchers to engage librarians in the review process to increase research quality [31, 32]. Developing a defined and structured service model to support increasing systematic review activities enabled us to address some common barriers and challenges to service provision. The two-tiered service model described here provides a framework that can be implemented or adapted by other libraries to help maximize the librarian’s time and expertise and improve research quality.

## SUPPLEMENTAL FILE

Appendix Work plan for knowledge synthesis librarian support
