# Supplementary material for: Building capacity for librarian support and addressing collaboration challenges by formalizing library systematic review services
Source: J Med Libr Assoc. 2019 Jul 1;107(3):411–9. doi: 10.5195/jmla.2019.443 (PMC6579587; doi:10.5195/jmla.2019.443)
Supplement: Appendix [file jmla-107-411-s001.pdf]

JMLA Journal of the Medical Library Association

Supplemental content to
J Med Libr Assoc. Jul;107(3):dx.doi.org/10.5195/jmla.2019.443
www.jmla.mlanet.org
© McKeown, Ross-White 2019

Building capacity for librarian support and addressing collaboration challenges by formalizing library systematic review services

Sandra McKeown, MLIS; Amanda Ross-White, MLIS, AHIP

APPENDIX

Work plan for knowledge synthesis librarian support

To be discussed with a librarian

The Canadian Institutes of Health Research (CIHR) define knowledge synthesis as "the contextualization and integration of research findings of individual research studies within the larger body of knowledge on the topic." A synthesis must be reproducible and transparent in its methods and may synthesize qualitative or quantitative results.

PART A

Research team

Names: *Indicate project lead/lead author		
Department(s):		
Status: (Select all that apply)<br>*Indicate project lead/lead author	☐ Faculty<br>☐ Medical resident<br>☐ Medical student	☐ Graduate student<br>☐ Undergraduate student<br>☐ Other:
Type of project:<br>(Select all that apply)	☐ Publication<br>☐ Guideline development<br>☐ Course work<br>↳Name and number:	☐ Thesis/dissertation<br>☐ Grant application<br>☐ Other:
Project time frame:		
First meeting with librarian: _______/_______/_______<br>day / month / year		Librarian:

JMLA Journal of the Medical Library Association

SC

Supplemental content to
J Med Libr Assoc. Jul;107(3):dx.doi.org/10.5195/jmla.2019.443
www.jmla.mlanet.org
© McKeown, Ross-White 2019

Synthesis type

☐	Systematic review	Seeks to systematically search for, appraise, and synthesize quantitative and/or qualitative research evidence, often adhering to guidelines on the conduct of systematic reviews such as the PRISMA Statement. Aims for comprehensive searching. A systematic review of quantitative and qualitative research may be referred to as a mixed-methods review.
☐	Meta-analysis	Seeks to systematically search for, appraise, and synthesize quantitative studies using a technique that statistically combines the results to provide a more precise effect of the results. Aims for comprehensive searching.
☐	Scoping review	Preliminary assessment of potential size and scope of available research literature. Aims to identify nature and extent of research evidence (usually including ongoing research) without formal quality assessment.
☐	Other	Please specify:

Have you conducted this type of synthesis before?	☐ Yes ☐ No
To your knowledge, does a synthesis or protocol already exists for this topic?<br>If yes, please explain:	☐ Yes ☐ No ☐ Unsure

Research plan

Will a protocol be prepared for this synthesis?	☐ Yes ☐ No ☐ Unsure
If yes, do you plan to register or publish the protocol?<br>If yes, please specify where:	☐ Yes ☐ No ☐ Unsure

Research question and type

The following models may help identify the main concepts of your topic to develop an answerable question:<br>PICO or PECO: **P**atients/**P**opulation/**P**roblem, **I**ntervention or **E**xposure, **C**omparator, **O**utcome<br>PICo: **P**opulation, phenomenon of **I**nterest, **Co**ntext
**Research question:** (e.g., In patients with [P], does [I] in comparison to [C] result in [O]?)
☐ Intervention ☐ Exposure ☐ Diagnosis ☐ Prognosis ☐ Epidemiology ☐ Experience<br>☐ Other:

JMLA Journal of the Medical Library Association

SC
Supplemental content to
J Med Libr Assoc. Jul;107(3):dx.doi.org/10.5195/jmla.2019.443
www.jmla.mlanet.org
© McKeown, Ross-White 2019

Study parameters

Note: Study parameters not necessarily incorporated into the search strategy.

Population:	☐ N/A<br>☐ Human studies<br>☐ Human and animal studies<br>☐ Other:	☐ N/A<br>☐ All ages<br>☐ All child (0–18)<br>☐ All adults (18+)<br>☐ Other:
Study type:<br>(Select all that apply)	☐ Quantitative ☐ Qualitative ☐ Mixed-methods<br>Please specify, if appropriate (e.g., randomized controlled trial [RCTs], cohort studies, etc.):	
Publication type:<br>(Select all that apply)	☐ Peer-reviewed articles ☐ Dissertations/Theses ☐ Conference<br>☐ Other:	
Languages:	Note: Even if non-English studies will not be included in the data analysis, it is still recommended to search and screen all languages in order to acknowledge the extent of non-English research on the topic (as a limitation of the synthesis).<br>☐ All ☐ Other:	
Publication dates:	☐ All ☐ Other (provide date range and rationale):	
Do you already know of any studies that may be eligible for your synthesis?<br>If yes, please send electronically.		☐ Yes ☐ No

Review software and citation management

Will you be utilizing review software for this project (e.g., Covidence)?<br>If yes, please specify:	☐ Yes ☐ No
Will you be utilizing a citation manager for this project (e.g., EndNote, Mendeley, etc.)?<br>If yes, please specify:	☐ Yes ☐ No

JMLA Journal of the Medical Library Association

SC

Supplemental content to
J Med Libr Assoc. Jul;107(3):dx.doi.org/10.5195/jmla.2019.443
www.jmla.mlanet.org
© McKeown, Ross-White 2019

PART B (To be completed with a librarian)

Search methods for identifying eligible studies

Databases:	☐ Ovid MEDLINE OR ☐ PubMed<br>☐ Embase<br>☐ Cochrane Library (CENTRAL)<br>☐ PsycINFO<br>☐ CINAHL<br>☐ AMED<br>☐ Web of Science<br>☐ BIOSIS Previews	☐ LILACS (Latin America)<br>☐ ERIC<br>☐ Sociological Abstracts<br>☐ SPORTDiscus<br>☐ REHABDATA<br>☐ PILOTS<br>☐ Other:
Grey literature sources (for research not published as a journal article):	☐ N/A<br>☐ ProQuest Dissertations and Theses<br>☐ Canadian Electronic Library<br>☐ Google Scholar<br>☐ Google<br>☐ Google Custom Search (government info)<br>☐ OpenGrey	☐ Clinical trial registries<br>☐ ClinicalTrials.gov<br>☐ WHO: International Clinical Trials Registry Platform<br>☐ Other:<br>☐ Other:
Cited reference searching:	☐ N/A<br>☐ Google Scholar	☐ Web of Science<br>☐ Other:
Additional search methods:	☐ N/A<br>☐ Screening references of eligible studies<br>☐ Hand-searching specific journals<br>Please specify:	☐ Hand-searching specific conference proceedings. Please specify:<br>☐ Other:
Search update:	Anticipated date, if applicable:	

Notes

Journal of the Medical Library Association

Supplemental content to
J Med Libr Assoc. Jul;107(3):dx.doi.org/10.5195/jmla.2019.443
www.jmla.mlanet.org
© McKeown, Ross-White 2019

PART C (To be completed with a librarian)

Librarian support

Advisory consultation is available to all faculty, staff, and students. Additionally, collaboration may be available to faculty, or research teams that include faculty, at the discretion of the librarian based on considerations such as whether or not a protocol for the research exists, the proposed time frame for the project, etc.

Advisory consultation services

A librarian can advise on the following:	
A preliminary search to determine if a review or protocol on the same topic already exists	☐
Review question formulation	☐
Review steps	☐
Database or resource selection for specific topics	☐
Database- or resource-specific search methods and techniques	☐
Setting up search alerts for new publications	☐
Citation management or review software	☐
Search methods for locating grey literature	☐
Additional methods for locating studies (searching trial registries, hand-searching, cited reference searching, etc.)	☐
How to obtain full-text articles via Queen's University Library and interlibrary loan (ILL)	☐
How the search methods should be reported for transparency and reproducibility	☐

Collaboration services

Please note: The following levels of library support should be negotiated up front as to whether the librarian's contribution will be formally recognized through acknowledgment or coauthorship.

A librarian may agree to do the following:	
Conduct a preliminary search to determine if a review or protocol on the same topic already exists	☐
Develop and execute database- or resource-specific search strategies	☐
Set up search alerts for new publications	☐
Document database-specific search strategies for transparency and reproducibility	☐
Export search results into desired format (Excel spreadsheet, text or RIS file, etc.)	☐
Import search results to citation management or review software	☐
Assist with search methods for locating grey-literature	☐
Assist with additional methods for locating studies (e.g., cited reference searching)	☐
Remove duplicate search results	☐
Write up the search methods according to PRISMA or other appropriate guidelines	☐

JMLA Journal of the Medical Library Association

Supplemental content to
J Med Libr Assoc. Jul;107(3):dx.doi.org/10.5195/jmla.2019.443
www.jmla.mlanet.org
© McKeown, Ross-White 2019

Notes

Visit our LibGuide for more information: http://guides.library.queensu.ca/knowledge-syntheses.
